# Investigating Stress-Related Heart Rate Behavior and Rhythm in College Students Using Trend Analysis Methods

**DOI:** 10.3390/s26082391

**Published:** 2026-04-14

**Authors:** Samira Ziyadidegan, Amir Hossein Javid, Farzan Sasangohar

**Affiliations:** Department of Industrial and Systems Engineering, Texas A&M University, College Station, TX 77840, USA; samiraziyadg@tamu.edu (S.Z.);

**Keywords:** heart rate (HR), mobile health (mHealth), mental stress, time series analysis, wearable sensors

## Abstract

**Highlights:**

**What are the main findings?**

- Heart rate patterns associated with stress are more chaotic during the day and at the beginning of the academic semester.
- There are persistent correlations in the heart rate data and less regular, less predictable heart rate patterns and rhythms during stress events.

**What are the implications of the main findings?**

- Current stress monitoring tools and models heavily rely on heart rate variability.
- Trend analysis techniques, such as autocorrelation and detrended fluctuation analysis, show promise for documenting stress-induced cardiac behavior.

**Abstract:**

(1) Background: Recent studies indicated the prevalence of stress among students. The increased level of stress is concerning due to its association with cardiovascular diseases. This study examined stress within the academic setting and its effects on heart rate patterns, addressing a gap in analysis methods beyond heart rate variability. (2) Methods: The data were collected from 125 students at a large university in Texas who were highly likely to experience stress disorders. Students were asked to wear a smartwatch for the duration of an academic semester to report their stress events. (3) Results: A total of 1513 stress events were reported. The highest frequency of stress events was reported at the beginning of the week, particularly on Tuesdays, and mostly between 10 am and 6 pm. Results also showed significant increases in the number of significant lags, the number of peaks in autocorrelation plots, and the scaling exponent in DFA plots. This indicates persistent correlations in the heart rate data and less regular, less predictable heart rate patterns and rhythms than during non-stress moments. (4) Conclusions: Findings underscore the importance of using time series analysis to understand the complexities in heart rate rhythm associated with stress, with the potential to inform future stress monitoring capabilities.

## 1. Introduction

Stress is an emotional or psychological response to demanding or challenging circumstances and has become increasingly prevalent among college students [1,2]. The mounting evidence also suggests that college student stress is widespread internationally. For example, a study conducted among 500 university students (50.4% male and 49.6% female) in the Middle East found that 35.4% experienced moderate stress, 13.2% severe, and 2.8% extremely severe [3]. Findings from a study of 2548 university students in Germany showed that 25.1% reported high perceived stress levels, as measured by the Perceived Stress Scale Questionnaire [4]. Another study of 320 students from a midwestern university in the United States confirmed the prevalence of stress (33.9%) [5]. Academic burdens, worries about the future, exams, and students’ obligation to succeed in academia are among the stressors, acute or chronic, faced by students [6,7].

The connections between mental stress and various health issues, including hypertension and other cardiovascular diseases, have been well-documented in the literature [8,9,10,11]. A study conducted on 52 students at Johns Hopkins Medical School revealed that high stress levels due to final exams and the first few weeks of school adversely affected their cardiovascular health [12]. Hence, individuals who consistently experience high stress levels may have an increased risk of cardiovascular diseases [9]. This concerning association between mental stress and cardiovascular diseases emphasizes the importance of investigating the relationship between stress and cardiovascular behaviors, such as heart rate and rhythm, among students.

Previous research has employed various techniques to examine changes in cardiac activities during stress events. The recent trend uses machine learning models to detect and predict cardiac activity and behavioral alterations during stressful events [13,14,15,16,17,18]. The more traditional methods use signal processing and descriptive statistical methods, such as *t*-tests and ANOVA, or threshold-based models, such as Poincaré plots. Despite the promise shown by previous approaches, most of the investigations either analyzed averaged values (e.g., applying a *t*-test to averaged heart rates) or failed to account for the effects of other external factors on heart rate (e.g., exercise). Moreover, although machine learning techniques are capable of analyzing heart behaviors [16,19], they are often difficult to interpret and less effective than tie-series analysis in understanding and modeling temporal changes during stress events.

More importantly, while heart rate is commonly used as a physiological marker in stress analysis, relying solely on heart rate is not a robust approach. Heart rate is influenced by numerous non-stress-related factors, including physical activity, posture, respiration, circadian rhythms, and individual cardiovascular fitness, which can confound the interpretation of stress [20]. Consequently, changes in heart rate do not uniquely reflect autonomic nervous system responses to psychological stress [20]. In contrast, heart rhythm dynamics more directly represent the balance between sympathetic and parasympathetic regulation rather than its average values [17,21]. Beyond heart rate variability (HRV) metrics [17,22,23], stress-related autonomic changes also manifest in the temporal structure and long-range correlations of heart rhythm signals. Trend analysis methods such as autocorrelation and detrended fluctuation analysis (DFA) [24] are therefore important for characterizing persistence, complexity, and scaling behavior in cardiac dynamics under stress. However, these methods remain largely underutilized in existing stress analysis frameworks, which tend to focus on static or short-term features. This reveals a clear gap in current methodologies, where incorporating heart rhythm–based trend and correlation analyses could substantially improve the robustness and interpretability of stress detection models.

To address these gaps, our research employed trend analysis techniques, including autocorrelation and detrended fluctuation analyses, to investigate heart rate patterns and rhythms, enabling an in-depth understanding of how stress impacts heart rate behavior during stress events among students.

## 2. Materials and Methods

A naturalistic study was conducted in a large university in the Southern United States to collect self-reported stress data among college students during a recent academic semester. We leveraged a previously developed mobile mental health app, the Mental Health Evaluation and Lookout Program (mHELP) for College Students [25], to facilitate reporting of stress events (i.e., double-tapping on a smartwatch screen) and continuous heart rate data collection via smartwatches. The study was approved by the university’s Institutional Review Board (IRB2020-0162DCR) and complied with the American Psychological Association Code of Ethics and the Declaration of Helsinki. Every participant was fully informed and provided their consent before participating in the study.

### 2.1. Participants

Recruitment emails were sent to all students via the university’s bulk email system, including study details and participation requirements, and specified participation for those who experienced high stress or used the university’s mental health services. Students (either undergraduates or graduates) completed an intake form and were considered for participation if they scored seven or above on the General Anxiety Disorder (GAD-7) test [26], which indicates a high likelihood of having Generalized Anxiety Disorder and at least moderate anxiety symptoms. Only iPhone users were eligible for this study. After finishing the study, students received a $150 Amazon gift card.

### 2.2. Design

Students were introduced to the study through a virtual onboarding session. They became familiar with the study’s goals, how to self-report a stress event, and how to ensure consistent data collection (e.g., charging the watch before bed and wearing it continuously). Participants were provided with an Apple Watch (series 4 or 5, Apple Inc., Cupertino, CA, USA) with the mHELP app pre-installed and were asked to wear it continuously for 90 days (with the exception of bed-time charging). The mHELP platform included a data monitoring capability that automatically uploaded batch data to the cloud via a HIPAA-compliant Amazon Web Services (AWS) service. A team of researchers reviewed the data daily to maintain consistency and accuracy in the collection process. The mHELP app operated continuously and recorded students’ heart rates. Students could simply double-tap their watch faces to report a stress event whenever they were significantly under stress, as in other studies [27].

### 2.3. Analysis

#### 2.3.1. Data Description

During the data collection period, the watch sensors recorded students’ heart rate data, capturing the average heart rate over the last 60 s at a frequency of 1 Hz. Additionally, the exact timestamp for each reported stress event was recorded.

#### 2.3.2. Data Preprocessing

Data preprocessing was performed in Python 3.10. This involved addressing missing and error values and windowing the data. Any heart rate values below 60 bpm [28] or above the maximum heart rate calculated using the 220-age formula [29] were identified as erroneous artifacts and removed. This resulted in a removal of 6.2% of the overall dataset. A 20 min window was set for stress events, 10 min before and 10 min after the reported stress event, and any data beyond this 20 min timeframe around the stress events was categorized as non-stress or “normal,” grounded in similar published methodologies [27,30,31]. To balance the data and examine differences between normal and stress events, we extracted the same number of 20 min normal events for each participant.

#### 2.3.3. Descriptive Analysis

To study the frequency of stress events and examine their relationship with associated heart rate data, a descriptive analysis was conducted to reveal the distribution of heart rate and its trends across days of the week and times of day. The Mann–Kendall test, a nonparametric test, was then used to analyze and identify trends in data without assuming normality.

#### 2.3.4. Trend Analysis

To examine whether heart rate data exhibit distinct patterns and rhythms during normal and stress events, autocorrelation and detrended fluctuation analysis were performed.

Autocorrelation plot: An autocorrelation plot, also referred to as a correlogram or autocorrelation function (ACF) [24], is a graphical representation that illustrates the strength of the relationship between a value and its lags (previous values) within a given time series. Since the correlations are calculated between a value and the lagged ones in the same time series, they are called serial correlations or autocorrelations [24]. The horizontal axis of the graph shows the lags, and the vertical axis shows the autocorrelation values, ranging from −1 to 1. A positive autocorrelation indicates that the current value is similarly influenced by previous values, likely following their increases or decreases. On the other hand, a negative autocorrelation indicates an opposing relationship. A number near zero indicates a very small or insignificant amount of autocorrelation.

In the autocorrelation plot, significance can be determined at the 95% confidence interval. Thus, the number of significant lags and the number of peaks (i.e., local maxima in the autocorrelation) within the 95% confidence interval were identified and recorded. Then, a paired *t*-test, used to compare the means of two related groups, was conducted to evaluate whether normal and stress events exhibit statistically different patterns.

In this study, the stationarity of the data, which is essential for valid autocorrelation analysis, was checked using the Augmented Dickey–Fuller (ADF) test [32]. When the data were not stationary, the differentiating technique was applied to render them stationary [33]. In this study, since most of the data were nonstationary, a first-order differencing technique was applied to all participants’ data to make the data homogeneous.

Detrended Fluctuation Analysis plot: Detrended fluctuation analysis is a statistical method applied to examine long-range correlations within time-series data and is not strict on the stationarity of the data [34,35,36]. In this method, the time series data is first integrated by calculating the cumulative sum of deviations from the average. Next, this data is split into equal-length segments. Within each segment, a polynomial plot (which is first order for most of the cases) is applied to identify the local trend. This local trend is then subtracted from the integrated time series, yielding a detrended time series. Then, the root-mean-square fluctuation is calculated. These values are then plotted against different segment sizes on a logarithmic scale to create a DFA plot [37].

The DFA plot shows how fluctuations in the data change with varying segment lengths. The slope of the line in the DFA plot, also known as the scaling exponent (alpha), provides information about the correlations of the time series data. Higher alpha values show higher long-range correlations in the time series data. An Alpha near 1.0 indicates pink noise [38], i.e., self-correlation.

To further analyze DFA plots, the average alpha values for normal and stress events for each participant were extracted. Using the paired *t*-test, we assessed whether normal and stress events demonstrated statistically different long-range correlations.

## 3. Results

### 3.1. Descriptive Analysis Results

In the study, 125 students from Texas A&M University were initially enrolled. However, only 117 students (92 females and 23 males, 1 non-binary, and 1 preferred not to disclose) completed the study and reported stress moments. Participants ranged from 18 to 37 years old (M = 22.2, SD = 4.34) and had diverse educational backgrounds, including 80 undergraduate and 37 graduate students.

**Figure 1:**
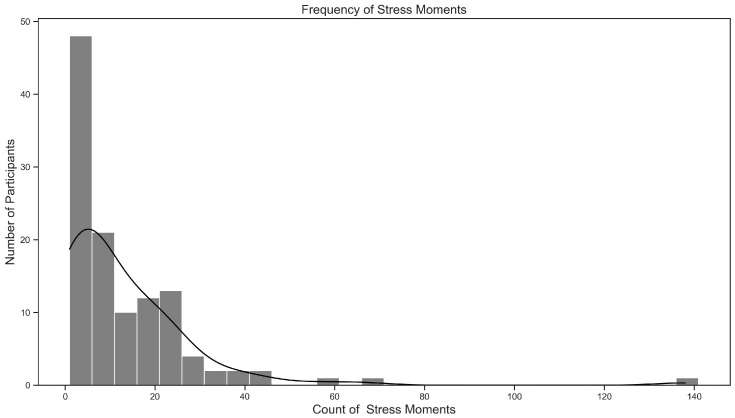
Frequency of the stress events.

**Figure 2:**
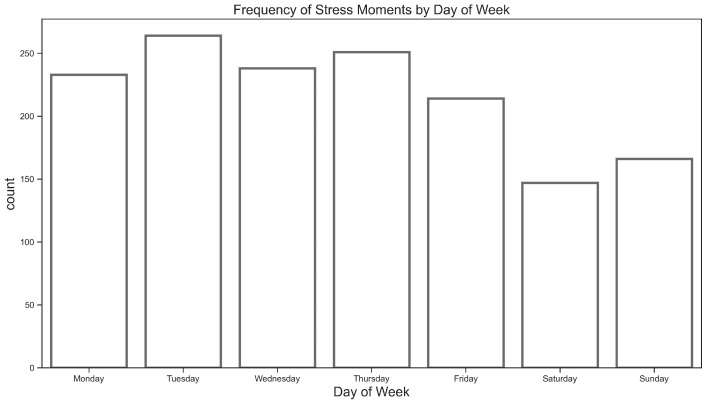
Frequency of stress moments by day of week.

A total of 1513 stress events were reported, averaging 12.9 per participant (standard deviation = 16.74; median = 7), as illustrated in Figure 1. Figure 2 shows the frequency of students’ stress events throughout the week. It shows that students reported the highest levels of stress at the early in the week, particularly on Tuesdays. Although school days showed significantly more stress events by average than weekends (1200/5 = 240 vs. 313/2 = 156.5, respectively), the Mann–Kendall Trend test did not detect a consistent trend from Monday to Sunday (*p*-value = 0.23).

**Figure 3:**
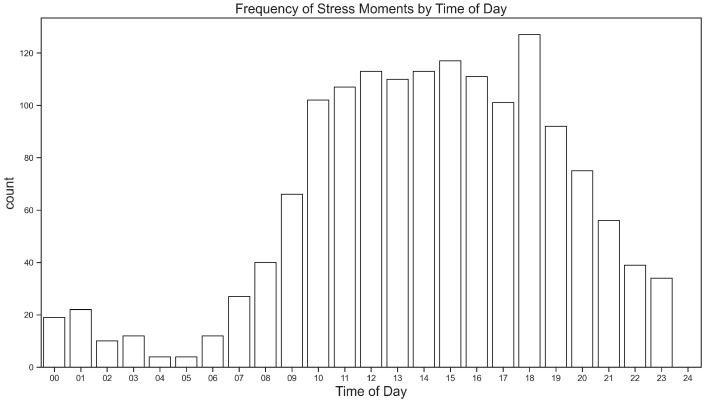
Frequency of stress moments by time of day.

Figure 3 shows the frequency of stress events by time of day. Findings indicated that most stress events (1001) occurred between 10 am and 6 pm and peaked at 6 pm. An analysis using the Mann–Kendall Trend Test identified an increasing trend from 6 am to 6 pm (*p*-value < 0.001) and a decreasing trend from 6 pm to 6 am (*p*-value < 0.001). The time period from 11 pm to 7 am depicted the fewest stress moments (only 7.27% of the total reported events).

**Figure 4:**
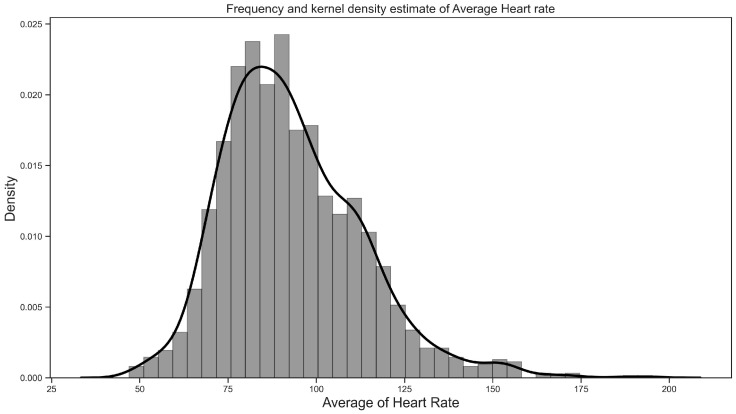
Frequency and kernel density estimate of the average heart rate.

Figure 4 displays the density distribution of heart rate associated with the reported stress events. Results revealed that the values ranged from 60 to 195 bpm (M = 93.2 bpm, SD = 19.64). It also shows that the majority of heart rate values during stress events are 90.53 (median = 90.53).

### 3.2. Trend Analysis Results

**Figure 5:**
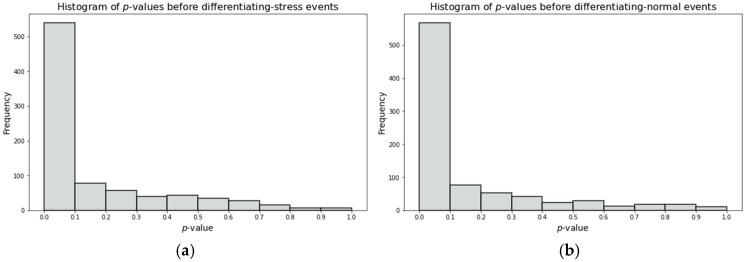
*p*-value for ADF test for (**a**) raw data during stress events, (**b**) raw data during normal events.

Figure 5 shows the *p*-values from the ADF test before first-order differencing for both stress and normal events. Results revealed that first-order differencing makes all the data stationary. 

**Figure 6:**
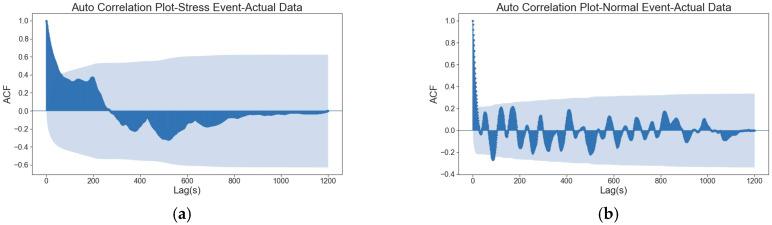
Autocorrelation plot for (**a**) raw data during stress events, (**b**) raw data during normal events. Light blue shaded regions indicate statistically significant areas.

Figure 6 illustrates the autocorrelation plot for a representative participant’s stress and normal events (one stress event and one normal event from a randomly selected participant) before and after differentiation. Light blue shaded regions indicate statistically significant areas. The figure shows that the correlation coefficient direction changed several times before reaching a lag of 200 s during normal events (Figure 6b). In contrast, the correlation coefficient remains positive for longer during the stress event (Figure 6a), and these changes are not observed in the stress event plot.

**Figure 7:**
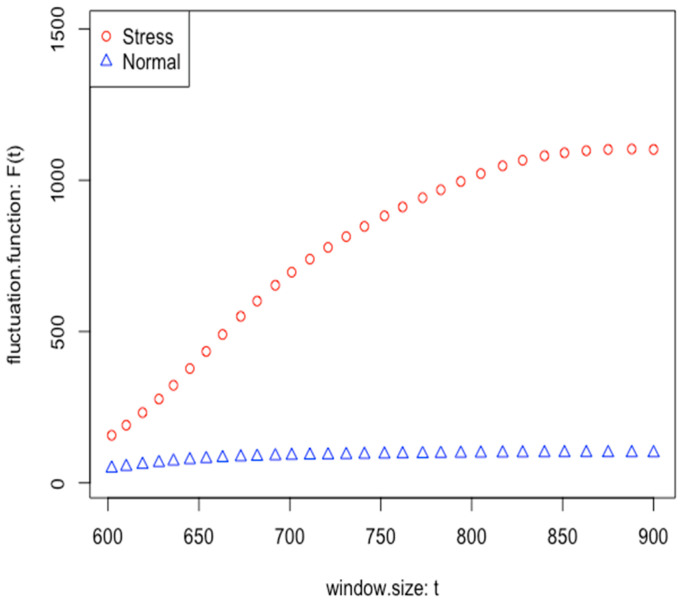
Detrended Fluctuation Analysis Plot comparing normal and stress events for a participant.

Figure 7 displays the DFA plot for 300 s samples (5 min after the reported stress moment) for a participant’s stress and normal events. The DFA values for stress events are significantly higher than those for normal events, especially over longer time intervals.

Table 1 presents a statistical comparison (paired *t*-test) between normal and stress events for autocorrelation analysis and DFA. The paired *t*-test was applied to the average values for each participant. The results indicate that the “number of significant lags” and “number of peaks” from the autocorrelation analysis and the “scaling exponent (alpha)” from DFA exhibit statistically significant differences between the two events.

## 4. Discussion

Recent studies indicate that students experience high levels of stress and feel overwhelmed by heavy academic responsibilities. This increased level of stress is concerning due to its association with cardiovascular diseases [39,40,41,42]. While the correlation between stress and cardiovascular functioning, and in particular heart rate has been studied [17,43,44,45], these studies overlook the temporal and rhythmic patterns of heart rate data.

In this study, we first documented the characteristics of stress events and the corresponding heart rate data among college students. Then, we employed time-series analysis to overcome issues associated with traditional descriptive analysis methods and the black-box nature of machine learning models [46]. Such methods, including autocorrelation and detrended fluctuation analysis, provide an efficient representation of the temporal characteristics of heart rate data. Employing such techniques makes the data easier to interpret and helps detect the temporal patterns and rhythms in heart rate data.

Frequency analysis of stress events indicated that stress was prevalent among students: it was higher during weekdays, with the highest frequency on Tuesday, compared to weekends. Results also showed that most stress events occurred during business hours and peaked at 6 pm. The reason is that students might be mentally overwhelmed and tired of attending classes, conducting projects, finishing assignments, and being involved in social interactions throughout the day. These stress patterns and frequency aligned with the typical academic schedules that students engaged in within the week and school days. Frequency analysis of heart rate data showed that during stress events, heart rates ranged from 60 to 195 bpm (M = 93.2 bpm, SD = 19.64 bpm) and had a median of 90.53 bpm. This shows that most stress events happen within a broad range of heart rate values and were not always limited to extreme values. These findings largely align with previous naturalistic studies, although these studies were conducted involving combat veterans [27,30].

Applying time-series techniques, such as autocorrelation and detrended fluctuation analysis (DFA), to heart rate data revealed specific patterns and trends during stress events. Autocorrelation revealed that normal heart rate data show several changes in correlation direction within a 200 s lag. On the other hand, the heart rate during stress events maintains a positive correlation for a longer period. Several features are extracted from autocorrelation and DFA plots and assessed to compare stress and normal events:

Number of significant lags: In autocorrelation plots, a higher number of significant lags was detected during stress events than during non-stress events for both actual and differentiated data. These findings suggest the persistence of stress effects on heart rate activity. In fact, this stochastic behavior lasted, on average, about ~27 s (752.67–725.28) during stress events. This cardiovascular response to stress is intervened by the autonomic nervous system, which initiates physical and mental responses (the fight-or-flight response) and releases Cortisol hormones that remain activated for a period of time [47,48,49].

Number of peaks: The number of peaks in autocorrelation plots of actual and differentiated data was significantly higher for normal events than for stress events. As autocorrelation plot shows how much the signal resemble itself, more fluctuations (a greater number of peaks) show that future values depend less on the past values in a consistent way. This finding suggests that heart rates exhibit irregular, unpredictable patterns during stress events [27]. This finding is aligned with previous research that used similar methods [27].

Scaling Exponent (alpha): The DFA plots indicated a significantly higher scaling exponent for stress events than for normal events. A higher scaling exponent during stress events indicates stronger long-range correlations, suggesting more persistent changes in heart rate after stress events. This result aligned with the higher number of significant lags observed in the autocorrelation analysis under stress.

While heart rate variability was not measured and studied in this study, these findings may be broadly consistent with the body of literature using heart rate variability to document manifestations of stress on heart rate patterns. For example, Mohammadi et al. demonstrated that specific heart rate variability measurements did not return to their initial baseline levels even after the recovery period [50]. Similarly, a study conducted by Held et al. revealed that individuals with anxiety disorders consistently display lower heart rate variability compared to healthy individuals under resting conditions [51].

### Gaps and Limitations

This study had notable limitations. One primary concern is the reliability of the self-reported stress events. Due to the subjective nature of stress perception, there is a potential for inconsistency in participants’ reports. Furthermore, this study did not consider variables such as caffeine consumption, sleep quality, and personal lifestyle choices. Also, chronic anxiety has not been considered in this study. These factors can affect both heart rate and stress levels. Additionally, minor changes to heart rate calculations due to artifacts such as movement artifacts might add error to the Apple watch collected data. Movement. In future research, we will aim to address these limitations by collecting more data or by designing a study that accounts for external factors. This will enhance the reliability of the findings.

## 5. Conclusions

Significant increases in the number of significant lags, the number of peaks in autocorrelation plots, and the scaling exponent in our study investigated the stress experienced by students and examined their heart rate rhythms over time. The study found that stress levels were higher on weekdays, especially from 10 am to 6 pm, underscoring the impact of academic tasks and the burden on students’ stress. During these stressful moments, the average heart rate was 93.2 bpm, which is within the normal range. Additionally, time-series analysis techniques, such as autocorrelation and Detrended Fluctuation Analysis (DFA) plots, revealed that normal and stress heart rate rhythms and patterns were clearly distinguished.

Results also showed significant increases in the number of significant lags, the number of peaks in autocorrelation plots, and the scaling exponent in DFA plots. This indicates persistent correlations in the heart rate data and less regular heart rate rhythms during stressful events. These findings underscore the importance of using time-series analysis and nonlinear features extracted from heart rate data to understand the complexities of stress-related heart rate dynamics. The features studied can be considered in enhancing stress detection models that currently rely on heart rate behavior as the main input. The increased lags and elevated scaling exponent in the stress dataset showed complex stress responses. Using time-series analysis methods can help understand the detailed patterns in heart rate data related to stress.

It should be note that this study does not cover short-term variability and the values extracted from the plots are estimated on temporally smoothed heart rate variabilities. Therefore, they cannot be reflected short-time variability (RR intervals).

## Figures and Tables

**Table 1:** Statistical Comparison of Extracted Metrics from Autocorrelation and DFA Plots for Normal and Stress Events (* denotes statistical significance *p* < 0.05).

Plot	Parameter	Event	Paired *t*-Statistic (Differentiated)	*p*-Value (Differentiated)	Average (Actual)	Average (Differentiated)
Autocorrelation Plot	Number of significant lags	Normal	3.5896	<0.001 *	725.28	41.80
		Stress			752.67	43.57
	Number of peaks	Normal	2.9379	0.004 *	11.72	370.03
		Stress			10.33	358.65
DFA Plot	Scaling Exponent (Alpha)	Normal	4.0456	<0.001 *	0.61	-
		Stress			0.63	-

## Data Availability

Data are contained within the article.
